# Synthesis and antibacterial properties of a novel magnetic nanocomposite prepared from spent pickling liquors and polyguanidine

**DOI:** 10.1039/c8ra03096k

**Published:** 2018-05-30

**Authors:** Dung T. Nguyen, Lan T. Pham, Ha T. T. Le, Minh X. Vu, Hanh T. M. Le, Huong T. M. Le, Nam H. Pham, Le T. Lu

**Affiliations:** Institute for Tropical Technology, Vietnam Academy of Science and Technology, Hanoi Hoang Quoc Viet str. 18, Cau Giay Hanoi Vietnam ndung@itt.vast.vn ltlu@itt.vast.vn; Graduate University of Science and Technology, Vietnam Academy of Science and Technology Hoang Quoc Viet str. 18, Cau Giay Hanoi Vietnam; Institute for Technology of Radioactive and Rare Elements, Vietnam Atomic Energy Institute Lang Ha 48, Dong Da Hanoi Vietnam; Institute Materials Sciences, Vietnam Academy of Science and Technology, Hanoi Hoang Quoc Viet str. 18, Cau Giay Hanoi Vietnam

## Abstract

Magnetic nanoparticles have received much interest for their application in wastewater treatment because of their easy retrieval and reuse. However, the methods used to synthesise high saturation magnetization magnetic nanoparticles require expensive and pure precursors. In the current study, we explore the potential for using spent pickling liquor, a wastewater solution from steel factories, as the iron precursor for preparing iron oxide nanoparticles. Here, magnetic Fe_3_O_4_ nanoparticles were synthesized *via* the oxidation–precipitation of spent pickling liquors using a saturated solution of calcium hydroxide at room temperature. The Fe_3_O_4_ nanoparticles were then modified with antibacterial polyguanidine to form a nanocomposite. It was found that monodisperse magnetic Fe_3_O_4_ nanoparticles with a size in the range 20–30 nm and a high saturation magnetization value of 73.9 emu g^−1^ were synthesised. The Fe_3_O_4_ nanoparticles were successfully encapsulated with polyguanidine to form an Fe_3_O_4_/polyguanidine nanocomposite. FT-IR and TGA analysis results indicated the presence of the polymer on the Fe_3_O_4_ surface and the polymer content in the nanocomposite was about 15% (w/w). The Fe_3_O_4_/polyguanidine nanocomposite exhibited strong antibacterial activity against *Escherichia coli* (*E. coli*), demonstrating its potential for use in disinfecting wastewater.

## Abstract 2

An Fe_3_O_4_/polyguanidine nanocomposite with strong antibacterial activity was prepared from Fe_3_O_4_ nanoparticles, using spent pickling liquors as the iron source, which were then encapsulated with polyguanidine.

## Graphical abstract

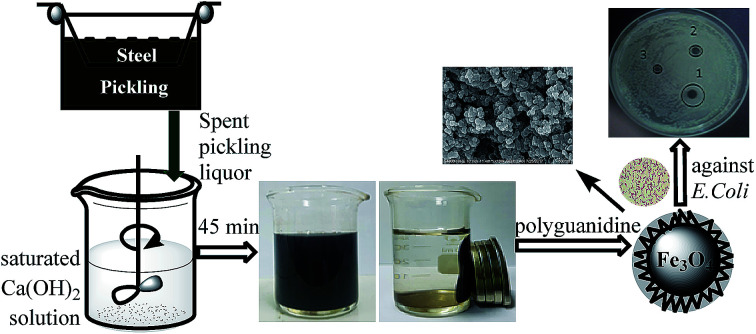

## 1. Introduction

In recent years, magnetic materials have attracted intensive interest due to their potential applications in various fields, including biomedicine,^1,2^ information storage,^3^ catalysts,^4^ and environmental treatment.^5–8^ Magnetic nanoparticles are promising for industrial scale wastewater treatment due to their high adsorption capacity, easy separation and enhanced stability. Magnetic nanoparticles can be produced using a variety of synthesis methods and can also be modified with different agents to form nanocomposites for the aforementioned applications.

In metal processing industries, iron and steel parts are often pickled in hydrochloric or sulfuric acid to remove the oxide layer from their surface before undergoing any further processing such as electroplating, metal finishing *etc.* The pickle solutions are gradually contaminated with dissolved metals through usage. As the metal concentration increases, the free acid concentration decreases and the pickling efficiency drops. To retain the high pickling efficiency, fresh concentrated acid is added from time to time to rejuvenate the bath but eventually it becomes spent and must be discarded. The waste generated by metal pickling industries is identified as an environmentally hazardous material.

Due to the high concentration of iron, spent pickling liquors (SPL) are possibly a cheap source of raw material for the production of iron materials. The conventional methods for SPL regeneration and metal recovery have been discussed in a recent detailed review.^9^ Generally, the regeneration of SPL is carried out by oxidizing its dissolved iron chloride (FeCl_2_) in a fluidized bed reactor at about 800 °C to form ferric oxide (Fe_2_O_3_) and gaseous hydrochloric acid. This is a relatively expensive process and it still produces an environmental pollutant, gaseous hydrochloric acid. In addition, the Fe_2_O_3_ recovered product has low economic value. Thus, it is important to develop a process that can produce a more valuable product from SPL with regard to both environmental considerations and processing cost.

For wastewater disinfection, most of the treatment methods involve chemicals and nanomaterials such as chlorine, ozone or Ag nanoparticles. These chemicals and materials possess high antibacterial activity. However, they exhibit some disadvantages: chlorine may harm the environment by forming toxic by-products such as trihalomethanes and haloacetic acids, which are potentially carcinogenic;^10,11^ ozone has no residual effects but it is sensitive to the presence of organic matters and can produce unknown toxic products;^12^ Ag nanoparticles may also have negative effects on the environment and human health.^13,14^ Hence, the development of safe antibacterial agents with high recovery or recycling ability has attracted considerable research interest in recent years.

In the last decade, guanidine-based polymers have attracted considerable attention as antibacterial materials due to their excellent biocide efficiency against a wide range of microorganisms. Zhang *et al.* have tested the antimicrobial activity of some polymeric guanidine and biguanidine salts against 10 bacterial and fungal species, and the determined MIC was not larger than 200 μg mL^−1^.^15^ Polyethylene hexamethylene biguanidine has been demonstrated by Wigdahl *et al.* to be a promising microbial candidate for anti-HIV compounds.^16^ In addition, polyguanidines have good thermal stability, low corrosive activity and lower toxicity for humans than the currently used disinfectants.^17^ However, the high water solubility restricts their potential application in some cases. There have been some studies reported recently about polyguanidine immobilization on different materials to form nonleaching antibacterial composites. Polyguanidine was used as a grafting agent on polyamide,^18^ polysulfone^19^ or cellulose membranes.^20^ Antibacterial experiments indicated a clearly enhanced anti-biofouling performance of the modified filtration membranes. Recently, polyguanidine was also investigated for use in graphene oxide functionalization, in the presence of polyethylene glycol for dispersion improvement. The as-prepared composite material demonstrated an enhanced antibacterial activity against both Gram-negative bacteria *Escherichia coli* and Gram-positive bacteria *Staphylococcus aureus*.^21^

In our current study, we combine polyguanidine and Fe_3_O_4_ magnetic nanoparticles to construct Fe_3_O_4_/polyguanidine nanocomposites possessing unique properties such as easy magnetic separation and anti-microbial activity that can be exploited in water purification applications. To the best of our knowledge, this antibacterial Fe_3_O_4_/polyguanidine magnetic nanocomposite has been synthesized for the first time *via* the oxidation–precipitation of SPL and modification with polyguanidine. The chemical and phase structures of the product were characterized, and the magnetic properties and antibacterial activity of the sample against *E. coli* were evaluated.

## 2. Materials and method

### 2.1. Chemicals

Samples of chloride pickling liquors (pH 0.1–0.2, total iron 151.2 g L^−1^, trace amounts of other heavy metals, *e.g.* Mn 26.7 mg L^−1^, Cr 16.9 mg L^−1^ and Cu 9.3 mg L^−1^) were collected from Hoa Phat Steel Factory, Vietnam. Calcium hydroxide (Ca(OH)_2_) of reagent grade was received from Xilong Scientific Co., China. Polyguanidine (Biopag-D) was ordered from FitoLine Co., Russia. All chemicals were used as received without further purification.

### 2.2. Preparation of magnetic nanocomposites

Fe_3_O_4_ nanoparticles were synthesized *via* an oxidation–precipitation process using spent pickling liquors according to our recent work.^22^ Briefly, 4.5 mL SPL was added drop-wise into a beaker containing 600 mL of saturated Ca(OH)_2_ solution at room temperature. The solution was stirred vigorously in air for about 45 min. The black precipitate of the Fe_3_O_4_ was separated using an external magnet, and was then washed with distilled water until the pH reached 7.

For the preparation of the Fe_3_O_4_/polyguanidine nanocomposite, 2 g purified Fe_3_O_4_ nanoparticles was added into 20 mL aqueous polyguanidine solution, 25% (w/w). The mixture was sonicated for 5 min and then stirred mildly for 6 h. The black precipitate was collected using a magnet, and was subsequently rinsed with distilled water and ethanol before being dried at 40 °C in a vacuum oven.

### 2.3. Characterisation

A field emission scanning electron microscope (FE-SEM, HITACHI S-4800) equipped with an energy dispersive X-ray spectroscope (EDX) was used to examine the morphology and elemental composition of Fe_3_O_4_ and the Fe_3_O_4_/polyguanidine nanocomposite. X-ray diffraction (XRD) patterns of the synthesized samples were taken with a Siemens/Bruker D5005 X-ray powder diffractometer over a 2*θ* range from 10° to 70°, using Cu K_α_ radiation (*λ* = 0.154 nm) with a step size of 0.03. Fourier transform infrared (FT-IR) spectra were recorded on a Thermo Scientific Nicolet iS10 FT-IR Spectrometer with Diamond ATR. Thermogravimetric analysis (TGA) was performed with a NETZSCH TG 209 F1 Libra instrument, from 25 °C to 600 °C in air (heating rate 10 °C min^−1^). The magnetic properties of the samples were studied using a vibrating sample magnetometer (VSM, DMS 800, Quantum Design, Inc.). Saturation magnetization (*M*_s_) and coercive field (*H*_c_) were measured at room temperature with the magnetic field ranging from −10 to 10 kOe.

### 2.4. Antibacterial tests

The disk diffusion method was used to evaluate the antibacterial activity of Fe_3_O_4_ and the Fe_3_O_4_/polyguanidine nanocomposite against *E. coli* (ATCC 8739). Mueller–Hinton agar was used as the growth medium. Briefly, 8 mm sterile disks impregnated with the magnetic nanoparticles (0.5, 1.7 and 5.0 mg mL^−1^) were placed on an *E. coli* cultured agar plate. After being incubated for 24 h at 37 °C, the diameter of the inhibition zone was determined.

## 3. Results and discussion

### 3.1. The influence of synthesis conditions on the magnetic properties

The direct oxidation–precipitation of the SPL in the saturated Ca(OH)_2_ solution produced magnetic nanoparticles. In the current study, we investigated the influence of synthesis conditions, including the speed of stirring and the volume of the precursor solution, on the morphology and magnetic properties of the nanoparticles. It was found that the magnetic properties of the material changed strongly with the variation of stirring speed and precursor concentration. The sample reached the highest *M*_s_ value of 74 emu g^−1^ at the stirring speed and precursor concentration of 400 rpm and 1.25 g L^−1^, respectively (Table 1). This sample was used for our further studies.

**Table 1:** The dependence of the saturation magnetization value (*M*_s_) on the stirring speed and precursor concentration

Speed^a^ (rpm)	*M*_s_ (emu g^−1^)	[Fe^2+^]^b^ (g L^−1^)	*M*_s_ (emu g^−1^)
200	40	0.5	21
400	73.9	1.0	58
600	63	1.25	74
800	22	1.5	67
		2.0	58
		2.5	54

a The samples were prepared at a precursor concentration of 1.25 g L^−1^.

b The samples were stirred at a speed of 400 rpm.

**Fig. 1:**
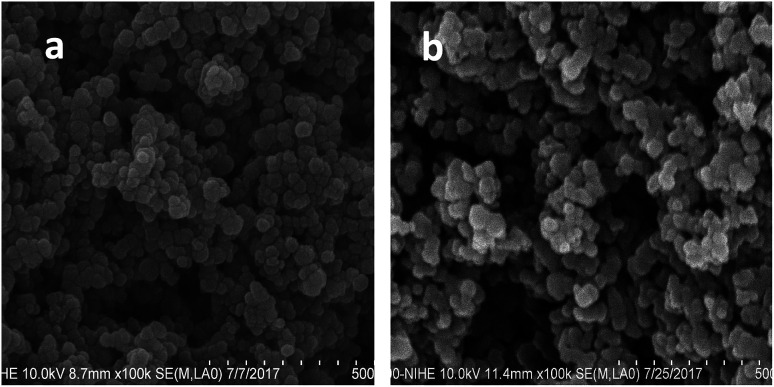
SEM images of (a) Fe_3_O_4_ and (b) the Fe_3_O_4_/polyguanidine nanocomposite.

For the antibacterial tests, the surface of the sample was modified with polyguanidine to improve its antibacterial activity. Fig. 1 shows the FE-SEM images of the sample before and after modification with the polymer. As shown in Fig. 1a, the iron oxide nanoparticles are fairly monodisperse and have a spherical shape with an average size of about 30 nm. A similar morphology was obtained for the nanocomposite (Fig. 1b).

**Fig. 2:**
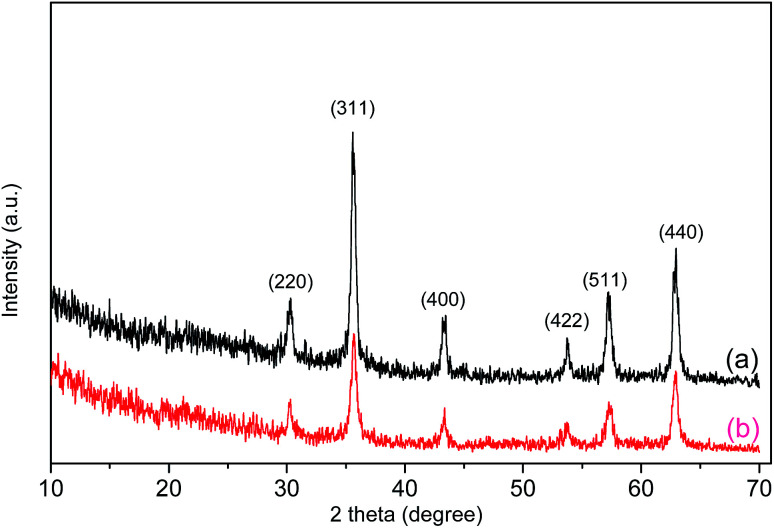
XRD patterns of Fe_3_O_4_ (a) and the Fe_3_O_4_/polyguanidine nanocomposite (b) prepared using the SPL precursor.

Fig. 2 shows the XRD patterns of Fe_3_O_4_ and the Fe_3_O_4_/polyguanidine nanocomposite. The samples show the characteristic diffraction peaks of the structural phase of spinel Fe_3_O_4_.^23^ This includes the typical peaks of (220), (311), (400), (422), (511) and (440) at the positions 30.2, 35.5, 43.3, 53.7, 57.2 and 62.9°, respectively. The absence of the characteristic diffraction peaks at (113), (210) or (213) of maghemite and hematite^24^ indicates that the nanoparticles synthesized from the spent pickling liquors in our work are single phase. After encapsulating with polyguanidine to form the nanocomposite, no signification change in the phase structure was identified.

**Fig. 3:**
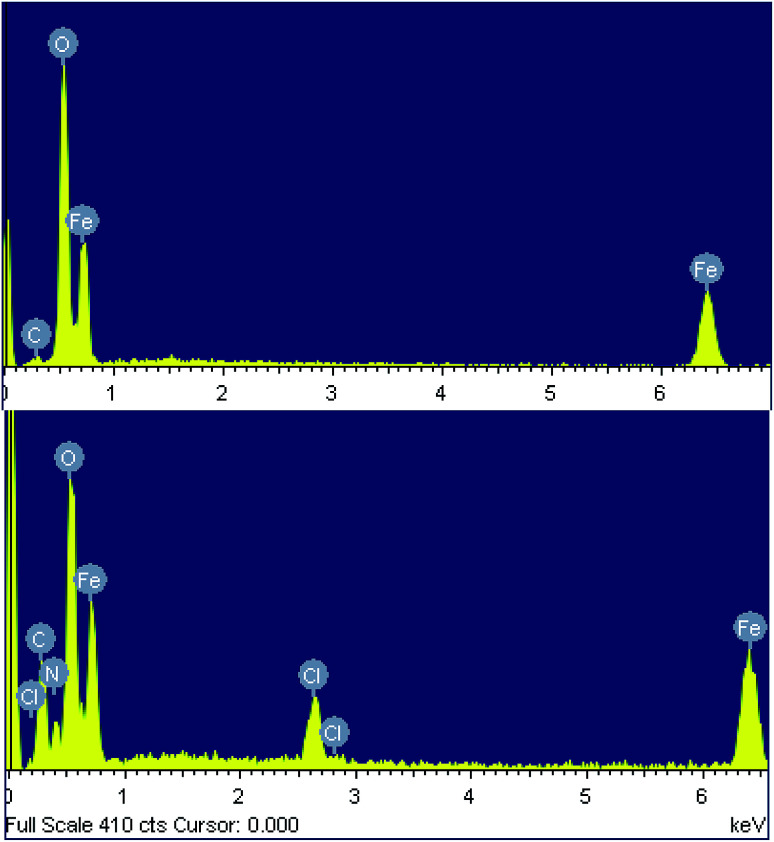
EDX of Fe_3_O_4_ (above) and Fe_3_O_4_/polyguanidine nanocomposite (below).

The chemical composition of both samples was determined using energy-dispersive X-ray spectroscopy (EDX). The EDX spectra of the nanoparticles synthesized from the spent pickling liquor demonstrate the presence of carbon (5.15% atomic), as well as Fe and O, indicating a little impurity of the product (Fig. 3a and Table 2). EDX data of the nanocomposite confirm the presence of polyguanidine by showing the coexistence of Fe, O, C, N and Cl in the spectrum (Fig. 3b and Table 2). From the EDX results, the polyguanidine content in the Fe_3_O_4_/polyguanidine is determined to be approximately 18% (w/w), which is similar to that determined using TGA (see the next section).

**Table 2:** The chemical composition of the Fe_3_O_4_ nanoparticles and Fe_3_O_4_/polyguanidine nanocomposite determined using EDX^a^

Element	Fe_3_O_4_		Fe_3_O_4_/polyguanidine	
	Weight (%)	Atomic (%)	Weight (%)	Atomic (%)
C K	2.46	5.15	15.74	29.69
O K	45.29	71.29	31.67	41.73
Fe K	52.25	23.56	44.01	20.09
Cl K			3.34	1.85
N K			5.24	6.62
Total	100.00		100.00	

a The EDX data are the average of nine measurement points.

### 3.2. FT-IR analysis

**Fig. 4:**
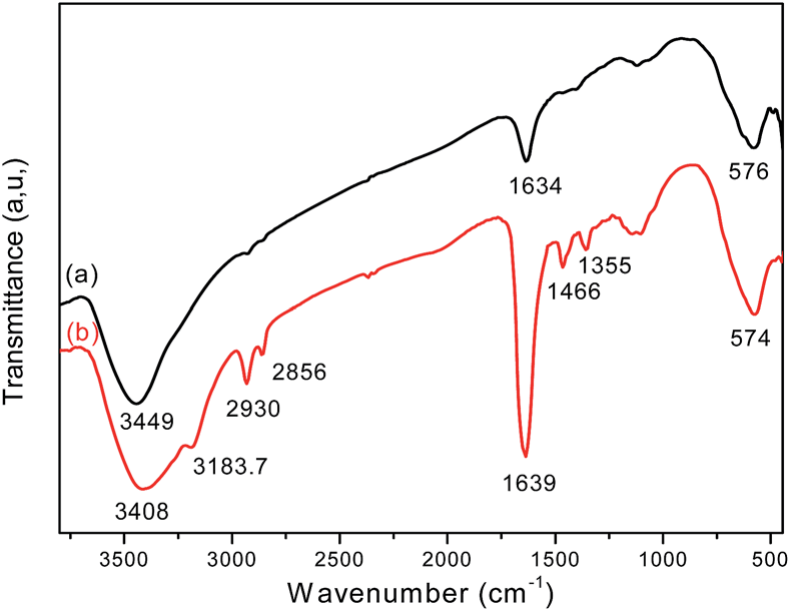
FT-IR spectra of (a) Fe_3_O_4_ and (b) the Fe_3_O_4_/polyguanidine nanocomposite.

FT-IR analysis provides direct evidence for the formation of Fe_3_O_4_ and the Fe_3_O_4_/polyguanidine nanocomposite (Fig. 4). The FT-IR spectrum of the sole Fe_3_O_4_ nanoparticles (curve a) shows the characteristic absorption band at 576 cm^−1^, which is attributed to the stretching vibration of the Fe–O bond of Fe_3_O_4_.^25,26^ Broad bands at around 3449 and 1634 cm^−1^ can also be seen, and these should be attributed respectively to the stretching and bending vibrations of the –OH groups on the surface of the nanoparticles.

In the spectrum of the Fe_3_O_4_/polyguanidine nanocomposite (curve b), in addition to the absorption peak at 574 cm^−1^ (which is due to the Fe–O stretching vibrations of the Fe_3_O_4_ nanoparticles), one can observe the characteristic absorption peaks of polyguanidine at around 3183.7 and 1466 cm^−1^, which could be related to the valence and bending vibrations of the amine group, respectively. The C–N stretching vibration band at 1355 cm^−1^ can also be observed, which is due to the existence of the secondary amine from polyguanidine. The peaks at around 2930 and 2865 cm^−1^ were assigned to the valence asymmetric and symmetric oscillations of the CH_2_ groups, respectively. The absorption peak at around 1639 cm^−1^ was very intensive, which was probably due to the overlap of the peak at 1634 cm^−1^ of Fe_3_O_4_ with the absorption peak of the C=N stretching vibration from polyguanidine. The broad peak at around 3449 cm^−1^ of Fe_3_O_4_ was also displaced to 3408 cm^−1^ because of the overlap with the peak of the N–H vibration from polyguanidine.^27^

### 3.3. TGA analysis

**Fig. 5:**
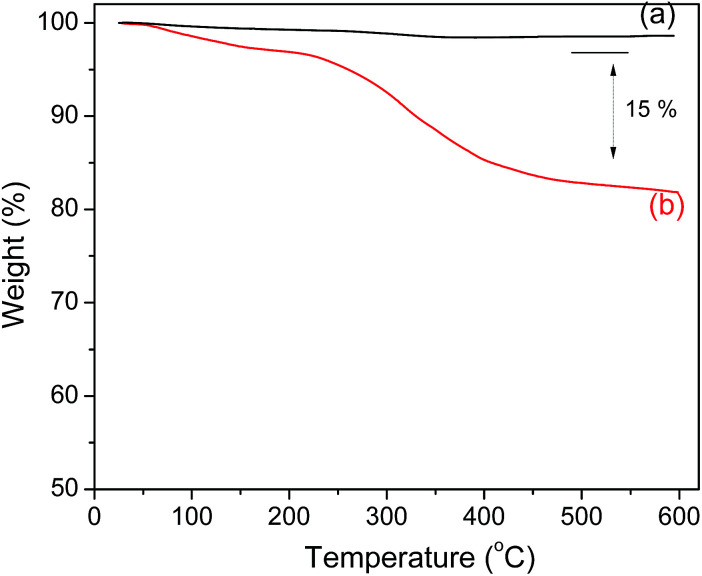
TGA curves of (a) the Fe_3_O_4_ nanoparticles and (b) the Fe_3_O_4_/polyguanidine nanocomposite.

The amount of polyguanidine on the Fe_3_O_4_ surface was determined using thermal analysis TGA. As shown in Fig. 5, the Fe_3_O_4_ nanoparticles exhibit very good thermal stability below 600 °C with insignificant weight loss in the TGA curve. On the other hand, the TGA curve of the nanocomposite indicates a low weight loss of about 5% starting from room temperature and continuing up to 200 °C due to dehydration, followed by a more obvious loss of weight starting at 250 and continuing up to 400 °C, which is related to the decomposition of polyguanidine.^15^ The amount of polyguanidine in the nanocomposite is about 15%.

### 3.4. Magnetic properties

**Fig. 6:**
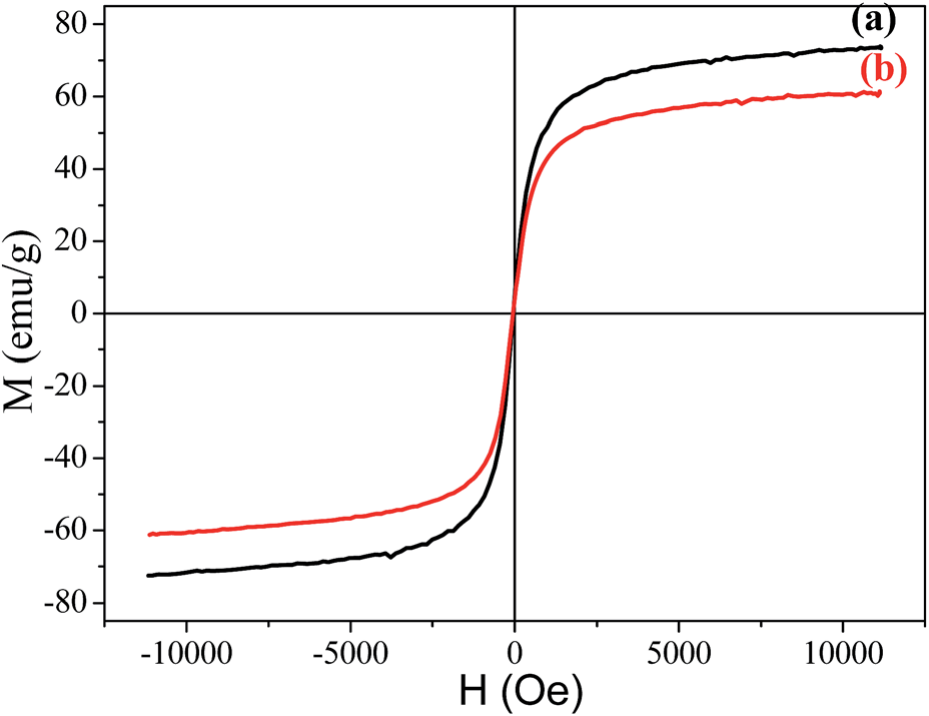
Magnetization curves of (a) Fe_3_O_4_ and (b) the Fe_3_O_4_/polyguanidine nanocomposite.

The magnetization hysteresis curves of Fe_3_O_4_ and the Fe_3_O_4_/polyguanidine nanocomposite were obtained using VSM magnetometry under a magnetic field ranging from −10 to 10 kOe at room temperature, and are shown in Fig. 6. These curves display the high saturation magnetization of both the pure and composite nanoparticles, with no coercivity or remanence, indicating that the samples are super-paramagnetic. As can be observed, the Fe_3_O_4_ nanoparticles synthesized from the SPL exhibit a high magnetic saturation (*M*_s_) value of 73.9 emu g^−1^. However, in the case of the Fe_3_O_4_/polyguanidine nanocomposite, the *M*_s_ value decreases to 61.2 emu g^−1^ due to the presence of the polymer layer.

### 3.5. Antibacterial activity

**Fig. 7:**
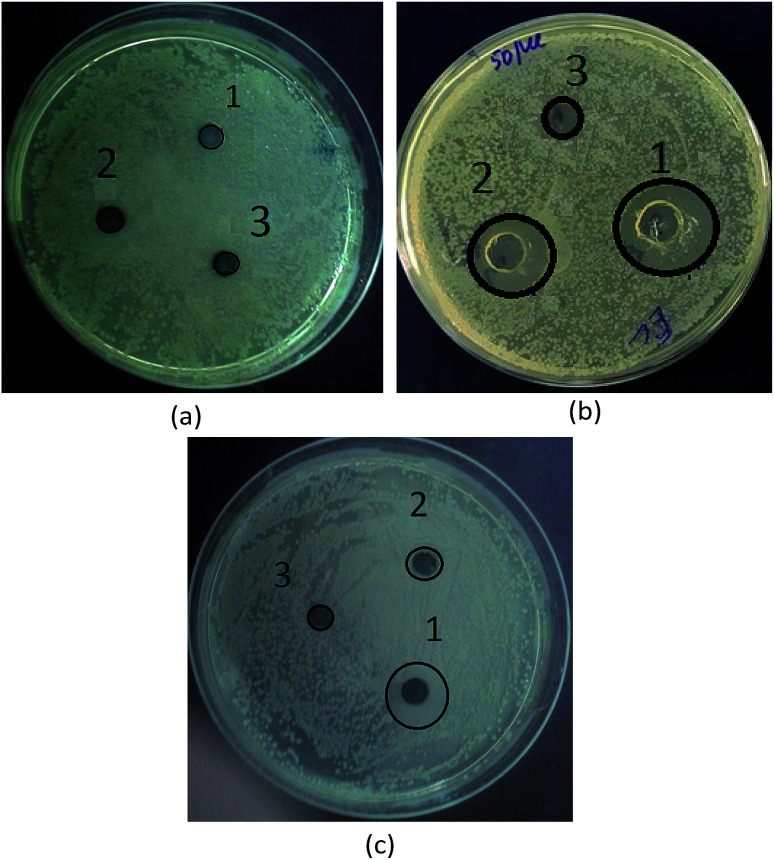
Photographs of the antibacterial test results of (a) Fe_3_O_4_, (b) polyguanidine and (c) the Fe_3_O_4_/polyguanidine nanocomposite at different sample concentrations: (1) 0.5, (2) 1.7 and (3) 5 mg mL^−1^.

In this work, the antibacterial activity of the as-prepared Fe_3_O_4_ and Fe_3_O_4_/polyguanidine nanocomposite at different concentrations of 5.0, 1.7 and 0.5 mg mL^−1^ was tested against *E. coli* using the agar diffusion method. Pure polyguanidine (Biopag-D) was also tested under similar conditions for comparison. The inhibition zones were determined and are shown in Table 3 and Fig. 7. It can be seen that sole polyguanidine with a concentration of 5.0 mg mL^−1^ presented an inhibition zone of 14 mm in diameter, which is almost the same as that reported by Grigor'eva *et al.*^27^ On the other hand, the Fe_3_O_4_ nanoparticles exhibited no antibacterial activity. The synthesized Fe_3_O_4_/polyguanidine nanocomposite had a relatively high antibacterial effect, presenting an inhibition zone of about 10 mm when the concentration was 5.0 mg mL^−1^. With a low content of polyguanidine in the nanocomposite (about 15%), the obtained results indicate a higher antibacterial efficiency of the Fe_3_O_4_/polyguanidine nanocomposite material than that of sole polyguanidine.

**Table 3:** The diameter of the inhibition zone (DIZ) surrounding the sample impregnated disks

Samples	Sample concentration (mg mL^−1^)	DIZ against *E. coli* (mm)
Fe_3_O_4_	5.0	0
	1.7	0
	0.5	0
Pure polyguanidine	5.0	14
	1.7	8
	0.5	0
Fe_3_O_4_/polyguanidine nanocomposite	5.0	10
	1.7	4
	0.5	0

## 4. Conclusion

In this study, a simple synthesis procedure was developed to prepare a novel magnetic Fe_3_O_4_/nanocomposite, using spent pickling liquors (SPL) and antibacterial polyguanidine. The analytical results from FE-SEM, EDX and FT-IR measurements indicated that a magnetite (Fe_3_O_4_)/polyguanidine nanocomposite with a size of about 30 nm and a saturation magnetization value of 62.1 emu g^−1^ was successfully synthesized *via* oxidation–precipitation in a calcium hydroxide solution of SPL under normal atmospheric pressure and room temperature. The amount of polyguanidine on the surface of the Fe_3_O_4_ nanoparticles was about 15% (w/w). This Fe_3_O_4_/polyguanidine nanocomposite exhibits a high antibacterial activity against *Escherichia coli*, and we believe that the obtained nanocomposite has the potential to be used in disinfection and biomedical applications. In our next work, the antibacterial activity of the Fe_3_O_4_/polyguanidine nanocomposite against various types of microorganism (including bacteria, viruses and fungi) will be investigated.

## Conflicts of interest

There are no conflicts to declare.

## Supplementary Material
